# Pediatric Trauma in The Netherlands: Incidence, Mechanism of Injury and In-Hospital Mortality

**DOI:** 10.1007/s00268-022-06852-y

**Published:** 2023-02-18

**Authors:** Christina Fylli, Inger B. Schipper, Pieta Krijnen

**Affiliations:** 1 Department of Surgery, Post Zone K6-R, P.O. Box 9600, 2300 RC Leiden, The Netherlands; 2 Department of Surgery, Leiden University Medical Center, Leiden, The Netherlands

## Abstract

**Background:**

In the Netherlands, there are no specialized or certified pediatric trauma centers, especially for severely injured children. National and regional agreements on centralization of pediatric trauma care are scarce. This study aims to describe the incidence, injury mechanism and in-hospital mortality of pediatric trauma in the Netherlands, as a prelude to the further organization of pediatric trauma care.

**Methods:**

A retrospective cohort analysis of data from the Dutch National Trauma Registry in 2009–2018, concerning all children (0–16 years) hospitalized due to injury in the Netherlands.

**Results:**

The annual number of admitted injured children increased from 8666 in 2009 to 13,367 in 2018. Domestic accidents were the most common cause of non-fatal injury (67.9%), especially in children aged 0–5 years (89.2%). Severe injury (injury severity score ≥  16) accounted for 2.5% and 74% of these patients were treated in level-1 trauma centers. In both deceased and surviving patients with severe injuries, head injuries were the most common (72.1% and 64.3%, respectively). In-hospital mortality after severe injury was 8.2%. Road-traffic accidents (RTAs) were the leading cause of death (46.5%).

**Conclusions:**

Domestic accidents are the most common cause of injury, especially in younger children, whereas RTAs are the lead cause of fatal injury. Severe pediatric trauma in the Netherlands is predominantly managed in level-1 trauma centers, where a multidisciplinary team of experts is available. Improving the numbers of severely injured patients primarily brought to level-1 trauma centers may help to further reduce mortality.

## Introduction

Since 2000, an average number of 80 children younger than 15 years old have died due to unintentional injuries each year in the Netherlands [1]. Regional studies showed that about 80% of the children that survive severe injuries in the Netherlands recover well, whereas 20% remain disabled to some extent after long-term follow-up [2, 3].

In 1999/2000, the Dutch trauma care system was regionalized with dedicated trauma centers appointed per region [4]. As a result, the injury-related in-hospital mortality rate in children aged 13–18 years has decreased over the period 1996–2006. However, no change was observed for children younger than 12 years. Injury prevention strategies, such as road safety regulations, have also led to a decrease in mortality related to some types of childhood injuries. In 2018, 19 children had died in a road-traffic accident in the Netherlands; on average 46 less fatalities per year than in the period 1996–2000 [5], due to improved road and traffic safety over the last decade.

Trauma care in children is fundamentally different from that in adults, because of their anatomical and physiological dissimilarities [6]. In the Netherlands, there are no specialized pediatric trauma centers and severely injured children are treated in any of the level-1 trauma centers. This may affect the outcome of care, especially for severely injured children, since it has been proven difficult to provide optimal pediatric trauma care without adequate experience [7].

The objective of this study is to describe the current epidemiology of pediatric trauma in the Netherlands, since an up-to-date overview is lacking. By analyzing data from the Dutch National Trauma Registry (DNTR), we aimed to determine the incidence of pediatric injury, and to identify trends in cause of injury, injury patterns and outcome of pediatric patients who were admitted to a hospital after trauma. Insight in these data will help to further optimize pediatric patient trauma care as well as trauma prevention on a national level in the Netherlands.

## Material and methods

### Study design and population

This retrospective cohort study analyzed data obtained from the DNTR, which was established in 2007 and includes information from all Dutch hospitals of patients admitted due to injury [8]. All pediatric patients aged 0 to 16 years who were admitted to one of the Dutch hospitals due to injury between January 1st, 2009, and December 31st, 2018, were included.

### Data collection

The primary outcome of this study was in-hospital mortality. Secondary outcomes were length of hospital stay (LOHS) and admittance to the intensive care unit (ICU). Other data included age, sex, type of hospital (trauma (level-1) vs. non-trauma center), injury characteristics (injury mechanism, injury type, abbreviated injury scale (AIS) score, injury severity score (ISS)) and vital signs on admission to the Emergency Department (ED) (Glasgow Coma Scale (GCS), respiratory rate (RR), systolic blood pressure (SBP)). In the DNTR, injuries are classified according to the AIS98 (up to 2015) and AIS08 (since 2015).

### Definitions

Patients with an ISS ≥ 16 and ISS ≥ 25 were considered as severely and critically injured, respectively [9]. A GCS of 13–15 corresponds with mild head injury, 9–12 with moderate head injury and 3–8 with severe head injury. Normal values for systolic blood pressure and respiratory rate vary per age group [10]. For each age category, values below normal were considered low and values above normal were considered high.

### Statistical analysis

To allow comparisons between age groups, children were divided in three age categories (0–5, 6–11, 12–16 years old). Categorical data were presented as number and percentage and compared using the Chi-square test. Injury severity scores were presented as median (interquartile range [IQR]) and compared using the Mann–Whitney test or Kruskal–Wallis test for independent samples. Additional analyses compared deceased and survived children and children treated in trauma vs. non-trauma centers. Missing data in this descriptive study were not accounted for in the analysis. The statistical analyses were performed in SPSS (IBM Corp. Released 2020. IBM SPSS Statistics for Windows, Version 27.0. Armonk, NY: IBM Corp).

No formal approval from the Institutional Review Boards was necessary, since only summarized data from the DNTR were obtained.

## Results

### Total admitted pediatric trauma population

**Fig. 1:**
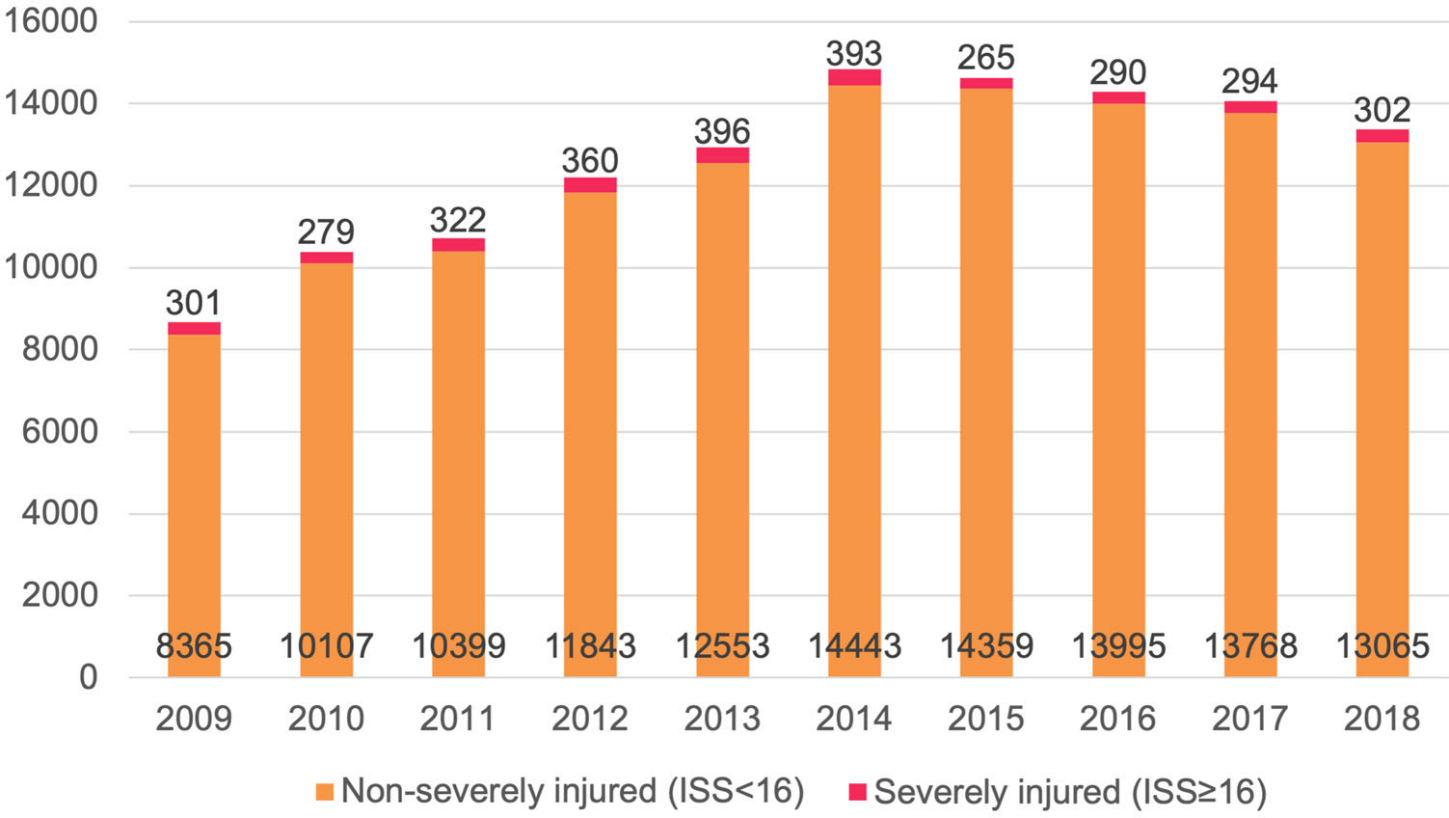
Annual number of hospitalizations due to injury in children aged 0–16 years in the Netherlands, 2009–2018, by injury severity score (ISS)

**Fig. 2:**
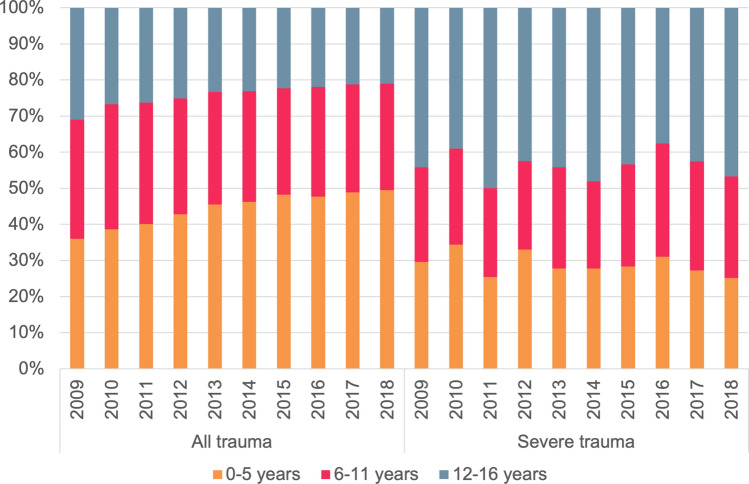
Age distribution of children admitted to the hospital due to injury in the Netherlands by calendar year, 2009–2018, for all pediatric trauma patients and for the subgroup of severe trauma (ISS ≥ 16)

The annual number of admitted injured children increased from 8666 in 2009 to 13,367 in 2018 (Fig. 1). During the study period, a total of 129,840 children were hospitalized due to injury, of whom 23.1% were treated in a level-1 trauma center (Table 1). The largest group was the 0–5-year-old group (44.9%). Over time, the proportion of hospitalized children aged 0–5 years increased and decreased for children aged 12–16 years (Fig. 2). Boys represented 58.8% of the population and formed the majority in all age categories (Table 1). Sex distribution remained unaltered over time.

**Table 1:** Characteristics of hospitalized children aged 0–16 years due to injury in the Netherlands in 2009–2018, per age group

	0–5 years (*n* = 58,296)	6–11 years (*n* = 40,595)	12–16 years (*n* = 30,949)	Total (*n* = 129,840)	*p* value	Missing n (%)
*Sex, n (%)*					<0.001	91 (0.1)
Male	32,536 (55.8)	23,417 (57.7)	20,364 (65.9)	76,317 (58.8)		
Female	25,726 (44.2)	17,156 (42.3)	10,550 (34.1)	53,432 (41.2)		
*Injury type, n (%)*					<0.001	9514 (7.3)
Blunt	52,637 (97.5)	36,607 (97.0)	27,541 (96.3)	116,785 (97.1)		
Penetrating	1343 (2.5)	1133 (3.0)	1065 (3.7)	3541 (2.9)		
*Mechanism of injury, n (%)*					<0.001	50,252 (38.7)
Domestic	34,258 (89.2)	14,780 (61.9)	5040 (29.1)	54,068 (67.9)		
Sport	630 (1.6)	5136 (21.5)	6024 (34.8)	11,790 (14.8)		
Road-traffic accident	2966 (7.7)	3480 (14.6)	5290 (30.6)	11,736 (14.7)		
Inflicted by others (violence)	156 (0.4)	162 (0.7)	542 (3.1)	860 (1.1)		
Self-harm/Suicide attempt	7 (0.0)	9 (0.0)	108 (0.6)	124 (0.2)		
Other	391 (1.0)	323 (1.4)	296 (1.7)	1010 (1.3)		
*Trauma center, n (%)*					<0.001	0 (0)
Trauma center	13,840 (23.7)	8595 (21.2)	7496 (24.2)	29,931 (23.1)		
Non-trauma center	44,456 (76.3)	32,000 (78.8)	23,453 (75.8)	99,909 (76.9)		
*Injury severity score, median (IQR)*						
2009–2014^a^	4 (1–6)	6 (4–9)	5 (4–9)	5 (3–8)	<0.001	3271 (4.5)
2015–2018^b^	2 (1–4)	4 (2–4)	4 (2–5)	4 (2–4)	<0.001	470 (0.8)
*Injury severity score, n (%)*						
2009–2014^a^					<0.001	3271 (4.5)
1–3	9040 (30.9)	3595 (15.8)	3168 (17.8)	15,803 (22.7)		
4–8	13,264 (45.4)	7965 (35.0)	7491 (42.1)	28,720 (41.2)		
9–15	6307 (21.6)	10,676 (46.9)	6204 (34.9)	23,187 (33.2)		
16–24	434 (1.5)	373 (1.6)	543 (3.1)	1350 (1.9)		
25 +	171 (0.6)	153 (0.7)	377 (2.1)	701 (1.0)		
2015–2018^b^					<0.001	470 (0.8)
1–3	18,903 (69.2)	5693 (33.8)	3951 (32.4)	28,547 (50.7)		
4–8	6197 (22.7)	9607 (57.0)	6322 (51.9)	22,126 (39.3)		
9–15	1887 (6.9)	1207 (7.2)	1420 (11.7)	4514 (8.0)		
16–24	183 (0.7)	226 (1.3)	279 (2.3)	688 (1.2)		
25 +	138 (0.5)	114 (0.7)	211 (1.7)	463 (0.8)		

*IQR* interquartile range

^a^Calculation based on injury coding according to AIS98

^b^Calculation based on injury coding according to AIS08

Injury mechanisms include road-traffic accidents (all accidents including a vehicle), self-harm accidents (including intentional intoxication and attempted suicide) and domestic accidents (accidents other than the above mechanisms of injury such as fall, accidental intoxication, accidental drowning and burns)

**Fig. 3:**
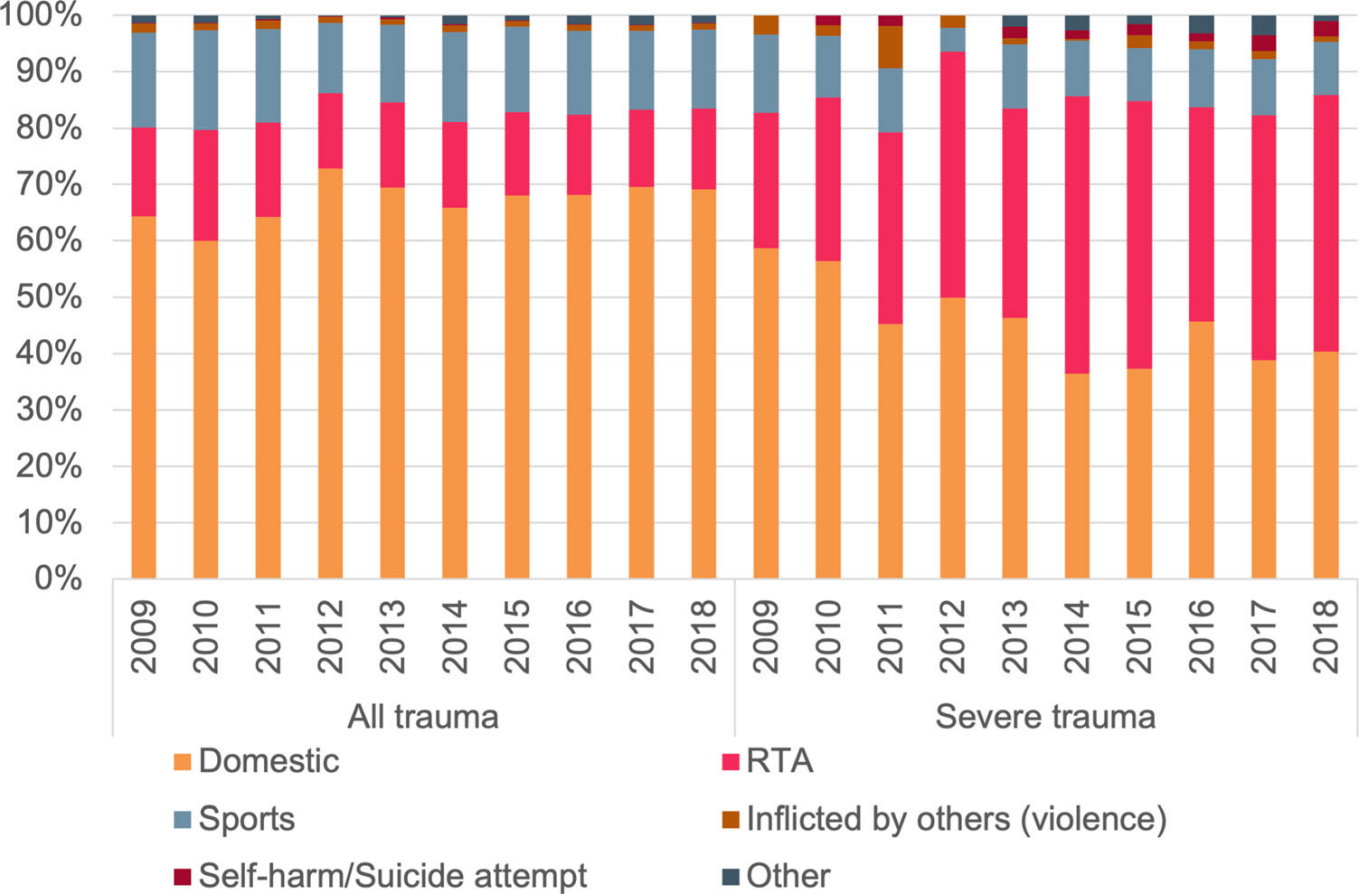
Distribution of injury mechanism in children admitted to the hospital due to injury in the Netherlands by calendar year, 2009–2018, for all pediatric trauma patients and for the subgroup of severe trauma (ISS ≥ 16)

Penetrating trauma was rare in each age group, but highest in the 12-16-year-old group (3.7%) (Table 1). Domestic accidents were the most common injury mechanism in children aged 0–11 years. The proportion of hospitalized children due to sport-related accidents and RTAs increased with age (Table 1). The distribution of other types of injury mechanisms was stable over time (Fig. 3).

Children aged 0–5 years were the least severely injured (Table 1). In 2015–2018, ISS was lower in all age categories compared to 2009–2014 due to the switch in the DNTR to AIS version 2008 in 2015 (Table 1).

### Severely injured (ISS ≥ 16) pediatric trauma population

Characteristics per age group and injury mechanism.

Severe injury accounted for 2.5% of all pediatric injury-related hospital admissions and children aged 12–16 years formed the largest age group in this population (44%) (Table 2). The majority in each age group were boys (62.8% in the total group). The age distribution of the severely injured children varied somewhat over time (Fig. 2).

**Table 2:** Patient and injury characteristics and clinical outcomes of severely injured children (injury severity score ≥ 16) aged 0–16 years in the Netherlands in 2009–2018, per age group

	0–5 years (*n* = 926)	6–11 years (*n* = 866)	12–16 years (*n* = 1409)	Total (*n* = 3202)	*p* value	Missing n (%)
*Sex, n (%)*					0.55	1 (0)
Male	568 (61.3)	547 (63.2)	895 (63.5)	2010 (62.8)		
Female	358 (38.7)	319 (36.8)	514 (36.5)	1191 (37.2)		
*Injury type, n (%)*					0.49	197 (6.2)
Blunt	841 (98.6)	804 (98.0)	1304 (97.9)	2949 (98.1)		
Penetrating	12 (1.4)	16 (2.0)	28 (2.1)	56 (1.9)		
*Mechanism of injury, n (%)*					<0.001	1468 (45.8)
Domestic	379 (74.5)	216 (44.8)	122 (16.4)	717 (41.3)		
Sport	4 (0.8)	50 (10.4)	117 (15.7)	171 (9.9)		
Road-traffic accident	105 (20.6)	200 (41.5)	444 (59.8)	749 (43.2)		
Inflicted by others (violence)	9 (1.8)	2 (0.4)	15 (2.0)	26 (1.5)		
Self-harm/Suicide Attempt	0 (0.0)	1 (0.2)	33 (4.4)	34 (2.0)		
Other	12 (2.4)	13 (2.7)	12 (1.6)	37 (2.1)		
*Trauma center, n (%)*					0.43	0 (0)
Trauma center	691 (74.6)	650 (75.1)	1027 (72.8)	2368 (74.0)		
Non-trauma center	235 (25.4)	216 (24.9)	383 (27.2)	834 (26.0)		
*Injury severity score, median (IQR)*						
2009-2014^a^	17 (16–25)	18 (16–25)	21 (17–27)	19 (16–29)	<0.001	0 (0)
2015-2018^b^	20 (17–25)	18 (17–25)	22 (17–26)	20 (17–25)	<0.001	0 (0)
*Injury severity score, n (%)*						
2009–2014^a^					<0.001	0 (0)
16–24	434 (71.7)	373 (70.9)	543 (59.0)	1350 (65.8)		
25 +	171 (28.3)	153 (29.1)	377 (41.0)	701 (34.2)		
2015−2018^b^					0.01	0 (0)
16–24	183 (57.0)	226 (66.5)	279 (56.9)	688 (59.8)		
25 +	138 (43.0)	114 (33.5)	211 (43.1)	463 (40.2)		
*Severe injury (AIS ≥ 3), n (%)*						
Head	672 (72.6)	549 (63.4)	861 (61.1)	2082 (65.0)	<0.001	0 (0)
Face	18 (1.9)	22 (2.5)	50 (3.5)	90 (2.8)	0.06	0 (0)
Neck	5 (0.5)	7 (0.8)	5 (0.4)	17 (0.5)	0.35	0 (0)
Thorax	128 (13.8)	156 (18.0)	450 (31.9)	734 (22.9)	<0.001	0 (0)
Abdomen	84 (9.1)	215 (24.8)	314 (22.3)	613 (19.1)	<0.001	0 (0)
Spine	17 (1.8)	20 (2.3)	90 (6.4)	127 (4.0)	<0.001	0 (0)
Upper extremities	13 (1.4)	34 (3.9)	86 (6.1)	133 (4.2)	<0.001	0 (0)
Lower extremities	46 (5.0)	92 (10.6)	204 (14.5)	342 (10.7)	<0.001	0 (0)
External	119 (12.9)	28 (3.2)	43 (3.0)	190 (5.9)	<0.001	0 (0)
*Glasgow Coma Scale in ED, n (%)*					0.01	485 (15.1)
13–15 (mild head injury)	409 (58.8)	488 (64.4)	763 (60.4)	1660 (61.1)		
9–12 (moderate head injury)	68 (9.8)	68 (9.0)	88 (7.0)	224 (8.2)		
3–8 (severe head injury)	218 (31.4)	202 (26.6)	413 (32.7)	833 (30.7)		
Respiratory rate^*^ in ED, n (%)					<0.001	977 (30.5)
Low	203 (35.6)	151 (25.0)	54 (5.1)	408 (18.3)		
Normal	233 (40.8)	284 (46.9)	752 (71.7)	1269 (57.0)		
High	135 (23.6)	170 (28.1)	243 (23.2)	548 (24.6)		
Systolic blood pressure^*^ in ED, n (%)					<0.001	464 (14.5)
Low	105 (15.5)	105 (13.8)	247 (19.0)	457 (16.7)		
Normal	211 (31.1)	421 (55.2)	752 (58.0)	1384 (50.5)		
High	362 (53.4)	237 (31.1)	298 (23.0)	897 (32.8)		
LOHS in days, median (range)	4 (1–109)	5 (1–101)	6 (1–109)	5 (1–109)	<0.001	193 (6.4)
ICU admittance, *n* (%)	472 (57.5)	483 (62.0)	849 (66.6)	1804 (62.8)	<0.001	328 (10.2)
In-hospital mortality, *n* (%)	86 (9.3)	53 (6.2)	123 (8.8)	262 (8.2)	0.03	19 (0.6)

Injury mechanisms include road-traffic accidents (all accidents including a vehicle), self-harm accidents (including intentional intoxication and attempted suicide) and domestic accidents (accidents other than the above mechanisms of injury such as fall, accidental intoxication, accidental drowning and burns)

*AIS* abbreviated injury scale; AIS ≥ 3: severe injury; *ED* emergency department; *ICU* intensive care unit; *IQR* interquartile range; LOHS: length of hospital stay

^a^Calculation based on injury coding according to AIS98

^b^Calculation based on injury coding according to AIS08

^*^Respiratory rate and systolic blood pressure values are age-specific and classified according to normal values per age group

RTAs were the leading injury mechanism in severely injured children aged 12–16 years (59.8%), while domestic accidents (41.3%) were most common in children 0–5 years (74.5%) and children aged 6–11 years (44.8%) (Table 2). Over time, the proportion of children with severe injury due to RTAs increased and there was a decrease in the proportion of children with severe injury due to domestic accidents (Fig. 3).

Children aged 12–16 years with severe injury had the highest median ISS: 21 (IQR 17–25) for the years 2009–2014 and 22 (IQR 17–26) for the years 2015–2018 (Table 2). More than one-third of the severely injured population was critically injured (ISS ≥ 25). This proportion differed significantly by age group (Table 2) and injury mechanism (Table 3).

**Table 3:** Injury characteristics of severely injured children (injury severity score ≥ 16) aged 0–16 years in the Netherlands in 2009–2018, by mechanism of injury

	Road-traffic accident (*n* = 749)	Domestic (*n* = 717)	Sport (*n* = 171)	Self-harm /Suicide attempt (*n* = 34)	Inflicted by others (violence) (*n* = 26)	Other (*n* = 37)	Total (*n* = 1734)	*p* value
*Severe injury (AIS ≥ 3), n (%)*								
Head	523 (69.8)	454 (63.3)	63 (36.8)	13 (38.2)	15 (57.7)	23 (62.2)	1091 (62.9)	< 0.001
Face	17 (2.3)	7 (1.0)	3 (1.8)	2 (5.9)	0 (0.0)	1 (2.7)	30 (1.7)	0.17
Neck	3 (0.4)	5 (0.7)	1 (0.6)	0 (0.0)	2 (7.7)	0 (0)	11 (0.6)	0.001
Thorax	232 (31.0)	85 (11.9)	26 (15.2)	11 (32.4)	8 (30.8)	8 (21.6)	370 (21.3)	< 0.001
Abdomen	159 (21.2)	96 (13.4)	72 (42.1)	2 (5.9)	6 (23.1)	4 (10.8)	339 (19.6)	< 0.001
Spine	41 (5.5)	25 (3.5)	8 (4.7)	5 (14.7)	0 (0)	0 (0)	79 (4.6)	0.01
Upper Extremities	18 (2.4)	17 (2.4)	3 (1.8)	5 (14.7)	0 (0)	0 (0)	43 (2.5)	< 0.001
Lower Extremities	133 (17.8)	31 (4.3)	8 (4.7)	6 (17.6)	0 (0)	3 (8.1)	181 (10.4)	< 0.00
External	10 (1.3)	110 (15.3)	4 (2.3)	14 (41.2)	0 (0)	7 (18.9)	145 (8.4)	< 0.001
*Injury severity score, median (IQR)*								
2009-2014^a^	22 (17–29)	17 (16–22)	17 (16–25)	24 (20–32)	24 (17–26)	29 (17–34)	20 (17–26)	< 0.001
2015-2018^b^	25 (17–33)	17 (16–25)	17 (16–21)	26 (18–32)	17 (17–26)	24 (16–25)	21 (16–28)	< 0.001
*Injury severity score, n (%)*								
2009-2014^a^								
16–24	146 (55.7)	202 (77.1)	44 (71.0)	5 (55.6)	5 (55.6)	4 (36.4)	406 (66.0)	< 0.001
25 +	116 (44.3)	60 (22.9)	18 (29.0)	4 (44.4)	4 (44.4)	7 (63.6)	209 (34.0)	
2015-2018^b^								
16–24	275 (56.5)	269 (59.1)	93 (85.3)	7 (28.0)	12 (70.6)	9 (34.6)	665 (59.4)	< 0.001
25 +	212 (43.5)	186 (40.9)	16 (14.7)	18 (72.0)	5 (29.4)	17 (65.4)	454 (40.6)	

Injury mechanisms include road-traffic accidents (all accidents including a vehicle), self-harm accidents (including intentional intoxication and attempted suicide) and domestic accidents (accidents other than the above mechanisms of injury such as fall, accidental intoxication, accidental drowning and burns)

*AIS* abbreviated injury scale; AIS ≥ 3: severe injury; *IQR* interquartile range

^a^Calculation based on injury coding according to AIS98

^b^Calculation based on injury coding according to AIS08

Head injuries were the most common severe injuries (AIS ≥ 3) (65%, Table 2). Severe injuries to the head and external injuries were the most frequent in children 0–5 years, whereas severe injuries to the face, thorax, abdomen, spine and extremities were more common in older children (Table 2). Severe head injury was present in the majority of children with severe injury after RTAs, domestic accidents and violent accidents (Table 3). About 20% of the severely injured children had severe injuries to the thorax and abdomen, mostly after RTAs, sport accidents (abdomen), self-harm (thorax), and violent accidents (Table 3).

Vital signs of severely injured children differed between the age groups. Age-specific low respiratory rates and high systolic blood pressure were more common in the youngest age group (Table 2). Children 12–16 years had the longest LOHS (median 6 days) and were admitted most often to the ICU (63%), whereas children 0–5 years had the shortest LOHS (median 4 days) and were less frequently admitted to the ICU (58%, Table 2).

### Trauma center admission

Seventy-four percent of the severely injured children were treated in a level-1 trauma center (Table 2). Severely injured children who were injured in an RTA, had a lower level of consciousness (GCS < 13), high RR, low SBP, higher ISS and severe injuries to the head, thorax, spine and lower extremities were more likely to be treated in a level-1 trauma center (Table 4). Consequently, severely injured children who were treated in a trauma center were longer hospitalized, were more frequently admitted to the ICU and had a higher mortality rate than those treated in a non-trauma center. Severely injured children who suffered a domestic or sport-related accident or had severe abdominal injury were more likely to be treated in a non-trauma center.

**Table 4:** Patient and injury characteristics and clinical outcomes of severely injured children (injury severity score ≥ 16) aged 0–16 years in the Netherlands in 2009–2018, by hospital level of trauma care

	Trauma center (*n* = 2368)	Non-trauma center (*n* = 834)	*p* value
*Age group, n (%)*			0.43
0–5 years	691 (29.2)	235 (28.2)	
6–11 years	650 (27.4)	216 (25.9)	
12–16 years	1027 (43.4)	383 (45.9)	
*Sex, n (%)*			0.40
Male	1497 (63.2)	513 (61.6)	
Female	871 (36.8)	320 (38.4)	
*Injury type, n (%)*			0.30
Blunt	2190 (98.0)	759 (98.6)	
Penetrating	45 (2.0)	11 (1.4)	
*Mechanism of injury, n (%)*			< 0.001
Domestic	520 (39.0)	197 (49.1)	
Sport	105 (7.9)	66 (16.5)	
Road-traffic accident	620 (46.5)	129 (32.2)	
Inflicted by others (violence)	24 (1.8)	2 (0.5)	
Self-harm/Attempted suicide	32 (2.4)	2 (0.5)	
Other	32 (2.4)	5 (1.2)	
*Injury severity score, median (IQR***)**			
2009-2014^a^	20 (17–26)	17 (16–22)	< 0.001
2015-2018^b^	24 (17–29)	17 (16–25)	< 0.001
*Injury severity score, n (%)*			
2009–2014^a^			< 0.001
16–24	887 (60.6)	463 (78.7)	
25 +	576 (39.4)	125 (21.3)	
2015–2018^b^			< 0.001
16–24	508 (56.1)	180 (73.2)	
25 +	397 (43.9)	66 (26.8)	
*Severe injury (AIS ≥ 3), n (%)*			
Head	1621 (68.5)	461 (55.3)	< 0.001
Face	73 (3.1)	17 (2.0)	0.12
Neck	16 (0.7)	1 (0.1)	0.06
Thorax	605 (25.5)	129 (15.5)	< 0.001
Abdomen	413 (17.4)	200 (24.0)	< 0.001
Spine	106 (4.5)	21 (2.5)	0.01
Upper extremities	97 (4.1)	36 (4.3)	0.78
Lower extremities	280 (11.8)	62 (7.4)	< 0.001
External	131 (5.5)	59 (7.1)	0.11
*Glasgow Coma Scale, n (%)*			< 0.001
13–15 (mild head injury)	1093 (53.1)	567 (86.0)	
9–12 (moderate head injury)	186 (9.0)	38 (5.8)	
3–8 (severe head injury)	779 (37.9)	54 (8.2)	
Respiratory rate^*^ in ED, n (%)			0.006
Low	301 (17.9)	107 (19.8)	
Normal	941 (55.8)	328 (60.7)	
High	443 (26.3)	105 (19.4)	
Systolic blood pressure^*^ in ED, n (%)			< 0.001
Low	375 (18.3)		
Normal	1025 (50.0)		
High	651 (31.7)		
LOHS, days in median (range)	6 (1–109)	2 (1–109)	< 0.001
ICU admittance, *n* (%)	1660 (74.2)	144 (22.6)	< 0.001
In-hospital mortality, *n* (%)	244 (10.3)	18 (2.2)	< 0.001

Injury mechanisms include road-traffic accidents (all accidents including a vehicle), self-harm accidents (including intentional intoxication and attempted suicide) and domestic accidents (accidents other than the above mechanisms of injury such as fall, accidental intoxication, accidental drowning and burns)

*AIS* abbreviated injury score; *AIS ≥ 3* severe injury; *ED* emergency department; *ICU* intensive care unit; *IQR* interquartile range; LOHS: length of hospital stay

^a^Calculation based on injury coding according to AIS98

^b^Calculation based on injury coding according to AIS08

^*^Respiratory rate and systolic blood pressure values are age-specific and classified according to normal values per age group

### In-hospital mortality

In the study period 2009–2018, 262 (8.2%) of the children with severe injuries died in the hospital (Table 2), mostly after an RTA or domestic accident (Table 5). Most of them were critically injured (ISS ≥ 25: 89.2% in the years 2009–2014; 93.5% in the years 2015–2018) and were in a coma (93.9% with GCS of 3–8). Nearly half of them were hypotensive on hospital admission (49.3%) (Table 5). Non-survivors more frequently had severe injuries to the head, neck, thorax and spine, as well as more severe external injuries (Table 5).

**Table 5:** Patient and injury characteristics of severely injured children (injury severity score ≥ 16) aged 0–16 years in the Netherlands in 2009–2018, by hospital survival

	Deceased (*n* = 262)	Survived (*n* = 2921)	*p* value
*Age group, n (%)*			0.03
0–5 years	86 (32.8)	837 (28.7)	
6–11 years	53 (20.2)	807 (27.6)	
12–16 years	123 (46.9)	1277 (43.7)	
*Sex, n (%)*			0.48
Male	170 (64.9)	1830 (62.7)	
Female	92 (35.1)	1090 (37.3)	
*Injury type, n (%)*			0.002
Blunt	238 (95.6)	2694 (98.4)	
Penetrating	11 (4.4)	44 (1.6)	
*Mechanism of injury, n (%)*			< 0.001
Domestic	54 (34.8)	661 (41.9)	
Sport	1 (0.6)	170 (10.8)	
Road-traffic accident	72 (46.5)	676 (42.9)	
Inflicted by others (violence)	6 (3.9)	20 (1.3)	
Self-harm/Attempted suicide	14 (9.0)	20 (1.3)	
Other	8 (5.2)	29 (1.8)	
*Injury severity score, median (IQR)*			
2009-2014^a^	34 (16–45)	18 (16–25)	< 0.001
2015-2018^b^	33 (25–43)	21 (16–26)	< 0.001
*Injury severity score, n (%)*			
2009–2014^a^			< 0.001
16–24	15 (10.8)	1322 (69.8)	
25 +	124 (89.2)	572 (30.2)	
2015–2018^b^			< 0.001
16–24	8 (6.5)	680 (66.2)	
25 +	115 (93.5)	347 (33.8)	
*Severe injury (AIS ≥ 3), n (%)*			
Head	189 (72.1)	1878 (64.3)	0.01
Face	10 (3.8)	77 (2.6)	0.26
Neck	5 (1.9)	12 (0.4)	0.001
Thorax	118 (45.0)	613 (21.0)	< 0.001
Abdomen	43 (16.4)	568 (19.4)	0.23
Spine	19 (7.3)	108 (3.7)	0.005
Upper Extremities	6 (2.3)	125 (4.3)	0.12
Lower Extremities	37 (14.1)	305 (10.4)	0.07
External	54 (20.6)	136 (4.7)	< 0.001
*Glasgow Coma Scale in ED, n (%)*			< 0.001
13–15 (mild head injury)	8 (3.5)	1641 (66.4)	
9–12 (moderate head injury)	6 (2.6)	216 (8.7)	
3–8 (severe head injury)	216 (93.9)	616 (24.9)	
Respiratory rate^*^ in ED, n (%)			< 0.001
Low	56 (32.0)	352 (17.3)	
Normal	83 (47.4)	1181 (57.9)	
High	36 (20.6)	506 (24.8)	
Systolic blood pressure^*^ in ED, n (%)			< 0.001
Low	103 (49.3)	353 (14.0)	
Normal	51 (24.4)	1324 (52.7)	
High	55 (26.3)	837 (33.3)	

Injury mechanisms include road-traffic accidents (all accidents including a vehicle), self-harm accidents (including intentional intoxication and attempted suicide) and domestic accidents (accidents other than the above mechanisms of injury such as fall, accidental intoxication, accidental drowning and burns)

*AIS* abbreviated injury scale; *AIS* ≥ 3 severe injury; *ED* emergency department; *IQR* interquartile range

^a^Calculation based on injury coding according to AIS98

^b^Calculation based on injury coding according to AIS08

^*^Respiratory rate and systolic blood pressure values are age-specific and classified according to normal values per age group

## Discussion

The annual number of registered pediatric injury-related hospitalizations in the Netherlands increased by nearly 60% from 2009 to 2014 and remained stable thereafter. This trend may predominantly be explained as an artifact, because the hospital participation rate in the DNTR increased from 64% in 2007 to 100% in 2015 [11]. Since 2015, the registration of patients admitted due to injury in the DNTR is near complete. Children aged 0–5 years accounted for an increasing part of all hospital admissions due to injury, which may be caused by the fact that young children are more likely to be admitted as a precaution than older ones [12].

Domestic accidents were the most common injury mechanism in children. Prevention of such injuries remains a global challenge [13]. Parent-focused strategies and product and environmental modifications have been proven effective in preventing childhood injuries [14, 15]. Modifications in the child’s environment such as the installment of stair gates, window guards and smoke alarms, childproofing caps on medication packaging, securing cabinets on walls and fencing a pool are some important first steps in home-injury prevention. Also, home visits by professionals can help improve the home environment and provide parents with information on safety equipment [16]. In the Netherlands, the Consumer and Safety Institute VeiligheidNL provides information to both parents and professionals regarding children’s safety and injury prevention [17]. In cooperation with the Ministry of Public Health, they organize trainings, campaigns and behavioral interventions with national, regional and local authorities. Active participation of local authorities to enhance the role of this organization may further help spreading awareness on childhood injury and prevention. Implementation of preventative measures is challenging, however. In addition to information campaigns, legal requirement and enforcement of prevention measures that are proven effective are necessary where possible.

In children aged 12–16 years, RTAs were the main cause of injury (60% of the cases). Children in this age group commonly bicycle unsupervised, making them more vulnerable in daily road traffic. Besides bicycling, use of light mopeds (two-wheeled vehicle) is quite common among Dutch 16-year-old children. Both modes of transportation do not require helmet use and make children at this age prone to head injury in RTAs. As observed in other studies, most RTA-related deaths in Dutch children involved children either as cyclists or pedestrians [18], while the majority of light-moped riders involved in fatal crashes between 2010 and 2015 were 16–17 years old [19].

Improvements in both road infrastructure and vehicle safety such as reducing the speed limit in areas where and at times when children and motorized traffic meet and introducing autonomous emergency braking systems with cyclist/pedestrian recognition will help to increase children’s safety in traffic [20]. For years, the obligatory use of bicycle helmets for Dutch children has been a matter of discussion. Bicycle helmets are still not obligatory because of the fear that this will strongly decrease bicycling, while this is good exercise for children. In our study, severe head injury was present in 65% of the total group of severely injured children and in 72% of the severely injured children that died in the hospital. Weijermars et al. [21] calculated that if all children under 12 years were to wear a bicycle helmet in the Netherlands, this could annually prevent up to 5 casualties and approximately 200 cases with survivable severe road-traffic injuries.

Even though the majority of the severely injured children were treated in a trauma center, 26% were treated in a non-trauma center, suggesting that further centralization of pediatric trauma in the Netherlands is necessary. Centralization of Dutch trauma care started in 1999 and since then treatment of severely injured patients including children has shifted to level-1 trauma centers, resulting in a regional reduction of 50% in crude mortality of trauma patients [22]. The goal of further centralization of care is not only to further reduce mortality but also to optimize outcomes of severely injured children who survive by offering timely and optimal diagnostics and treatment. The American College of Surgeons Committee on Trauma (ACS-COT) recommends a maximum undertriage rate of 5% in polytrauma patients [23], while in the Netherlands, this is 10% [22]. In pediatric trauma, undertriage rates often exceed 20%, which is mostly caused by differences in triage protocols [24, 25]. Currently, the Dutch National Protocol of Ambulance Services and Field Triage Decision Scheme triage protocols are used in pediatric field triage in the Netherlands. Recent studies revealed that both protocols cannot meet current sensitivity targets but also cannot accurately distinguish between low-risk and high-risk trauma patients [26, 27]. Accordingly, the development of a sensitive and child-specific triage protocol is needed in order to prevent undertriage in severely injured children.

Overall in-hospital mortality (0.2%) was low compared to other European countries and the USA (Spain, 0.5%; Norway, 1%; the USA, 1.2%; the UK, 3.7%; Switzerland, 5.5%; Denmark, 7.3%), and mortality after severe injury (8.2%) was comparable to mortality for similar patients in other countries (the USA, 3.2%; Spain, 12.8%; Germany, 13.4%; Switzerland, 17.5%) [28–35]. This suggests that the quality of early pediatric trauma care in the Netherlands is high, including short prehospital times, advanced trauma care at the accident site, designation of trauma centers, improvements in intensive care, assessing of all injured patients according to ATLS guidelines and ‘quick’ interhospital transfers [36].

There are some limitations to take into consideration when interpreting the results of this study. The first limitation is the missing data for some of the cases. Variables with the highest percentages of missing values were mechanism of injury (39% in the total group and 46% in the severely injured group) and physiological variables (GCS 15%, RR 30%, SBP 14% in the severely injured patients), while the remaining variables were complete for 90–100% of the cases (Tables 1–2). Missing values for mechanism of injury were considered to be missing at random. Physiological variables may be missing not at random, since values within the normal clinical range are more likely not to be reported in the medical files. We assume that the reasons for missingness of data were not different in the subgroups of patients that were described, so that Tables 1–3 offer a fair comparison of these subgroups. Heinänen et al. and Ali et al. reported a 97% and 61% case completeness of the Helsinki and Navarre (Spain) Trauma Registry, respectively [37, 38]. Registration of initial observations of the injured patients must be further improved in order to increase data quality and reliability of the results.

A second limitation is the update of the injury severity scoring tool, the AIS, from the 1998 to the 2005 version, which took place in 2015. According to Hsu et al. [39], the adaptation of the AIS-2005 version resulted in a significant reduction of severity of injury scores in the measurement of the same injuries. Since the aim of this study was to observe recent trends in the epidemiology of pediatric trauma, we included the years 2015–2018, for which the AIS-2005 version applied. By splitting the study period in two (2009–2014 and 2015–2018), we were able to present injury severity scores adapted to the AIS-2005 version separated from each other, in an attempt to minimize misleading outcomes of injury severity. Nonetheless, the results concerning injury severity should be interpreted with caution.

## Conclusions

Domestic accidents are a significant cause of injury, especially in younger children, and RTAs are the lead cause of fatal injury. Severe pediatric trauma in the Netherlands is predominantly managed in trauma centers, where a multidisciplinary team of experts is available. Improving the numbers of severely injured patients primarily brought to level-1 trauma centers may help further reduce mortality. Also, injury prevention programs should be directed toward increasing safety measures in traffic.
